# Chemical Composition and Biological Activities of *Chromolaena odorata* (L.) R.M.King & H.Rob. Essential Oils from Central Vietnam

**DOI:** 10.3390/molecules30173602

**Published:** 2025-09-03

**Authors:** Hoa Van Vo, Prabodh Satyal, Thuong Thanh Vo, Truc Thi-Thanh Le, An Thi-Giang Nguyen, Hien Thi Vu, Trung Thanh Nguyen, Hung Huy Nguyen, William N. Setzer

**Affiliations:** 1 Department of Pharmacy, Duy Tan University, 03 Quang Trung, Da Nang 550000, Vietnamthuongvoart@gmail.com (T.T.V.); lethithanhtruc132@gmail.com (T.T.-T.L.); 2 Aromatic Plant Research Center, 230 N 1200 E, Suite 100, Lehi, UT 84043, USA; psatyal@aromaticplant.org (P.S.); wsetzer@chemistry.uah.edu (W.N.S.); 3 Faculty of Biology, College of Education, Vinh University, 182 Le Duan, Vinh City 4300, Vietnam; nguyengianganbio@gmail.com; 4 Faculty of Hydrometeorology and Water resources, Ho Chi Minh City University of Natural Resources and Environment, Ho Chi Minh City 70000, Vietnam; vthien@hcmunre.edu.vn; 5 Center for Pharmaceutical Biotechnology, College of Medicine and Pharmacy, Duy Tan University, 03 Quang Trung, Danang 550000, Vietnam; trungnt@duytan.edu.vn; 6 Center for Advanced Chemistry, Institute of Research and Development, Duy Tan University, 03 Quang Trung, Da Nang 5000, Vietnam; 7 Department of Chemistry, University of Alabama in Huntsville, Huntsville, AL 35899, USA

**Keywords:** *Aedes* spp., *Culex* spp., intermediate host, biopesticide, antimicrobial

## Abstract

The chemical composition of leaf essential oil of the harmful invasive species *Chromolaena odorata* collected in Vietnam was analyzed by GC/MS and chiral GC. All three essential oil samples (O1, O2 and O3) in this study fell into chemotype I characterized by α-pinene/geigerene/germacrene D/(*E*)-β-caryophyllene from a total of six different chemotypes. Chemotype I demonstrated larvicidal effects against *Aedes aegypti* (Linnaeus, 1762), *Aedes albopictus Aedes albopictus* (Skuse, 1894), *Culex fuscocephala* (Theobald, 1907) and *Culex quinquefasciatus* (Say, 1823), with 24 h LC_50_ values ranging from 11.73 to 69.87 µg/mL. In contrast, its microemulsion formulation exhibited enhanced toxicity, yielding 24 h LC_50_ values between 11.16 and 32.43 µg/mL. This chemotype also showed repellent efficacy against *Ae. aegypti*, with protection times ranging from 70.75 to 122.7 min. Fumigant toxicity was observed against *Aedes aegypti*, with LC_50_ values of 40.27% at 0.5 h and 0.34% at 24 h. Molluscicidal activity was recorded with 48 h LC_50_ values between 3.82 and 54.38 µg/mL against *Indoplanorbis exustus* (Deshayes, 1833), *Pomacea canaliculate* (Lamarck, 1822), *Physa acuta* (Draparnaud, 1805). Additionally, the chemotype exhibited acetylcholinesterase inhibitory activity, with an IC_50_ value of 70.85 µg/mL. Antimicrobial potential was also demonstrated, with MIC values ranging from 2.0 to 128.0 µg/mL against *Enterococcus faecalis*, *Staphylococcus aureus*, *Bacillus cereus*, *Escherichia coli*, *Salmonella enterica*, and *Candida albicans*. The *C. odorata* essential oil can be considered as a potential bioresource for human health protection strategies.

## 1. Introduction

*Chromolaena odorata* (L.) R.M.King & H.Rob. is a long-lived shrub belonging to the Asteraceae family, originally native to the tropical and subtropical areas of Central and South America, including regions such as Mexico, the Caribbean, and Brazil. Due to a combination of anthropogenic factors—including global trade, tourism, transportation, and alterations in land use—and natural dispersal mechanisms—such as wind, water currents, runoff, and animal movement—the species has extended its range across Africa, Oceania, and parts of South and Southeast Asia. It is widely regarded as a highly invasive weed, particularly in tropical perennial crop systems such as coffee, cocoa, and citrus plantations [1]. *C. odorata* has widely colonized secondary forest habitats from northern to southeastern Vietnam, where its presence inhibits the regeneration and establishment of native plant species [2]. Despite being classified as a harmful invasive species, *C. odorata* is regarded in this study as a promising and readily available raw material for the development of essential oil-based products. These products have potential applications in health protection strategies, including biopesticides, antibiotics, and aromatherapy for supporting the treatment of Alzheimer’s disease. Several studies have reported that the essential oil yield of *C. odorata* ranges from approximately 0.85% to 1.03% [3,4]. This relatively high yield, combined with the plant’s availability, represents a significant advantage in terms of efficiency and cost-effectiveness for large-scale industrial production [5].

This study investigates the chemical makeup and biological activity of *C. odorata* essential oils and their potential to control three medically important mosquito species: *Ae. aegypti*, *Ae. albopictus*, and *Cx. quinquefasciatus*. *Aedes* mosquitoes are major carriers of arboviruses like dengue [6], Zika [7], yellow fever [8], and chikungunya [9,10,11], while *C. quinquefasciatus* transmits the filarial worm *Wuchereria bancrofti* [12] and has been linked to the spread of Rift Valley fever and possibly urban Zika transmission [13].

We also evaluated molluscicidal activity against freshwater snails as intermediate hosts for parasitic diseases. *I. exustus* transmits cattle schistosomiasis, human cercarial dermatitis, and liver flukes (*Fasciola hepatica*, *F. gigantica*) [14], as well as other trematodes like *Paramphistomum* and *Echinostoma* [15]. *P. acuta*, an invasive species, hosts parasites such as *Angiostrongylus cantonensis* [16], *Echinostoma revolutum* [17], *Posthodiplostomum minimum*, and *Hypoderaeum conoideum* [18]. *P. canaliculata* spreads *A. cantonensis* [19] and *Gnathostoma spinigerum* [20] while damaging rice crops [21].

This study also evaluated the antimicrobial activities against major bacterial and fungal species capable of multidrug resistance, which are listed as common causative agents of community- and hospital-acquired infections, including *E. faecalis*, *S. aureus*, *B. cereus*, *E. coli*, *P. aeruginosa*, *S. enterica*, and *C. albicans* [22,23,24].

Essential oils and their constituents have demonstrated potential neurological effects, including therapeutic roles in neurodegenerative disorders such as Alzheimer’s and Parkinson’s diseases [25]. Due to their low molecular weight and lipophilic nature, these compounds can efficiently cross the blood–brain barrier [25]. Furthermore, their volatility enables administration via inhalation, thereby bypassing hepatic metabolism, which could otherwise degrade bioactive compounds [26]. Therefore, in this study, we also evaluated the acetylcholinesterase (AChE) inhibitory activity of *C. odorata* essential oil.

## 2. Results and Discussion

### 2.1. Chemical Profiles of Essential Oils

The extraction yields of the three essential oil samples O1, O2 and O3 were 0.31, 0.32 and 0.30% (*w*/*w*) on fresh weight, respectively. Chemical compositions of *C. odorata* essential oils from Vietnam are presented in Table 1. These three essential oils have a similar chemical composition, with the main components being α-pinene (11.47–19.24%), germacrene D (11.67–15.12%), (*E*)-β-caryophyllene (9.56–11.24%) and geijerene (8.96–10.55%), followed by components β-pinene (3.95–7.50%), δ-cadinene (4.38–5.73%), caryophyllene oxide (4.40–5.33%) and α-copaene (4.02–5.26%).

**Figure 1:**
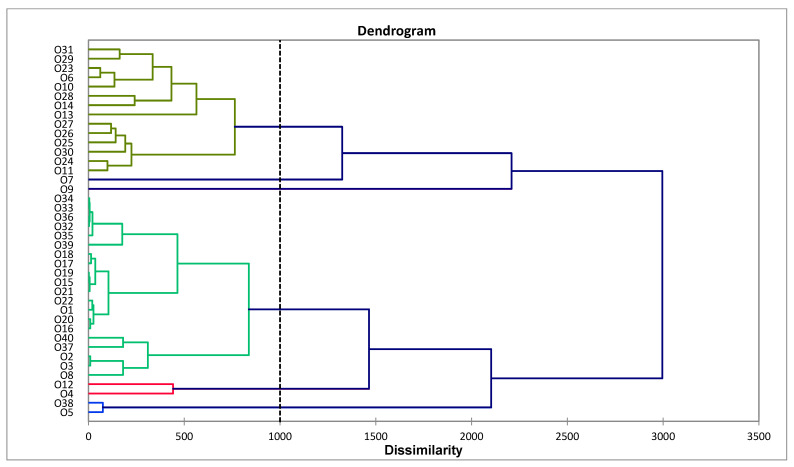
Dendrogram based on agglomerative hierarchical cluster analysis of chemical compositions of *Chromolaena odorata* essential oils. Principal component analysis of chemical compositions of *Chromolaena odorata* essential oils. Details on the components, full chemical composition, and collection locations of the essential oil samples corresponding to codes O1 to O40 are available in Supplementary Material Table S1.

**Figure 2:**
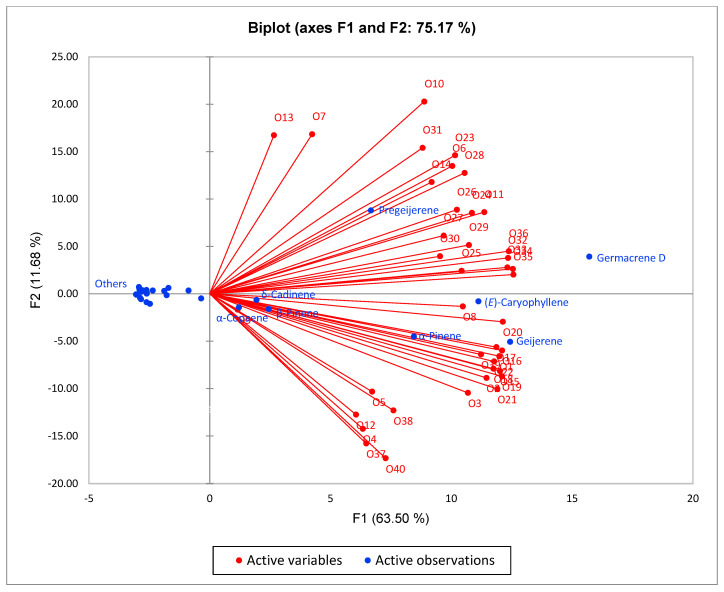
Principal component analysis of chemical compositions of *C. odorata* essential oils. O4: [27]; O5: [28]; O6: [29]; O7: [30]; O8: [31]; O9: [4]; O10: [32]; O11: [32]; O12: [3]; O13: [33]; O14: [33]; O15–O22: [34]; O23–O29: [35]; O30: [36]; O31: [37]; O32–O36: [38]; O37: [39]; O38–O39: [40]; and O40: [41]. Details on the components, full chemical composition, and collection locations of the essential oil samples corresponding to codes O1 to O40 are available in Supplementary Material Table S1.

In order to place the essential oil compositions of *C. odorata* from Vietnam in this study with essential oil compositions from other geographical locations reported in the literature, an agglomerative hierarchical cluster (AHC) analysis was carried out (Figure 1). The cluster analysis revealed at least six chemical clusters: (I) α-pinene/geigerene/germacrene D/(*E*)-β-caryophyllene, (II) geigerene, (III) α-pinene, (IV) pregeijerene/(*E*)-caryophyllene/germacrene D, (V) pregeijerene, and (VI) caryophyllene oxide. The samples from Vietnam that fall into cluster I are O1, O2, O3 and O39, while the two samples O4 and O38 fall into clusters II and III, respectively. A principal component analysis (PCA) was carried out to verify the AHC analysis (Figure 2), and it confirms the correlation of the samples from Vietnam with geigerene, germacrene D, and (*E*)-β-caryophyllene.

To our knowledge, this is the first time that the enantiomeric distribution of monoterpenes in *C. odorata* essential oils has been determined. The enantiomeric distribution of monoterpenes in *C. odorata* essential oils was similar among the three essential oil samples. However, when compared with essential oils of other Asteraceae species that have been analyzed by enantioselective GC-MS, there do not seem to be consistent trends in enantiomeric distributions (Table 2). Thus, for example, (+)-sabinene was the predominant enantiomer in *C. odorata* but was variable in other members of the family. (+)-β-pinene was dominant in *C. odorata* but is often the minor enantiomer is other members of the family (e.g., *Artemisia ludoviciana*, *Ambrosia acanthicarpa* [42], *Achillea millefolium* var. *occidentalis* [43], and *Ericameria nauseosa* [44]). Although (+)-limonene was the major enantiomer in *C. odorata*, the distribution seems to be variable in other members of the Asteraceae.

### 2.2. Characteristics of Microemulsion Formulas

**Figure 3:**
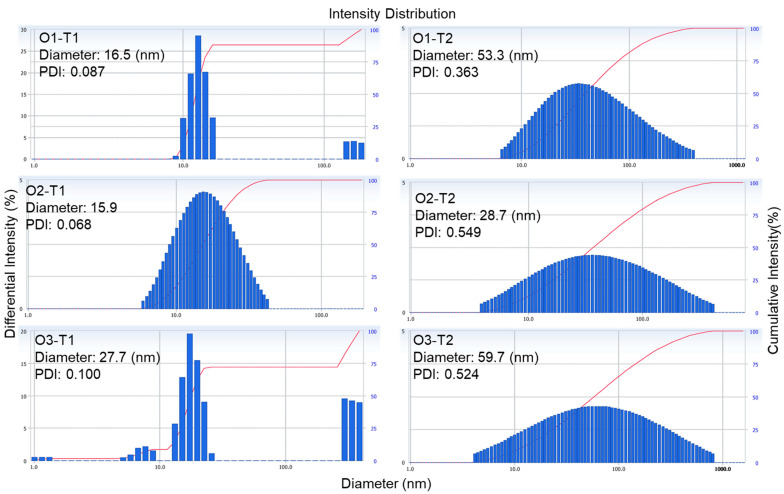
Dynamic light scattering (DLS) traces of microemulsions (MEs) at different timepoints (t1 and t30 days).

The microemulsion formulations exhibited physical parameters (particle size and PDI) ranging from 15.9 to 27.7 nm and 0.068 to 0.100 at T1 and in the range of 28.7–53.3 nm and 0.363–0.549 at T2, respectively (Figure 3).

The combination of alcohols and surfactants in microemulsion (ME) formulation is well established [45,46,47,48,49]. Short-chain alcohols reduce droplet size and improve oil solubilization in oil–water MEs [50]. Awad et al. reported uniform droplet formation in Tween 80/MCT emulsions at MCT concentrations below 1% [51].

### 2.3. Larvicidal Activity

**Figure 4:**
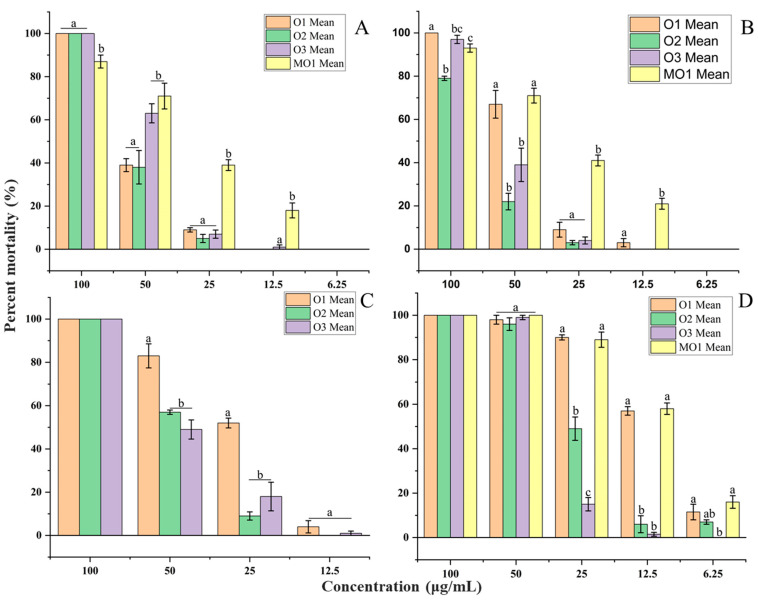
Mortality rate (%) of mosquito larvae at different concentrations of samples O1, O2, O3 and MO1 after 24 h of exposure, with mean ± SE. Values followed by the same letter (a–c) at each concentration are not statistically different at *p* < 0.05 as measured by Tukey’s test. (**A**) *Aedes aegypti*. (**B**) *Aedes albopictus*. (**C**) *Culex quinquefasciatus*. (**D**) *Culex fuscocephala*.

**Figure 5:**
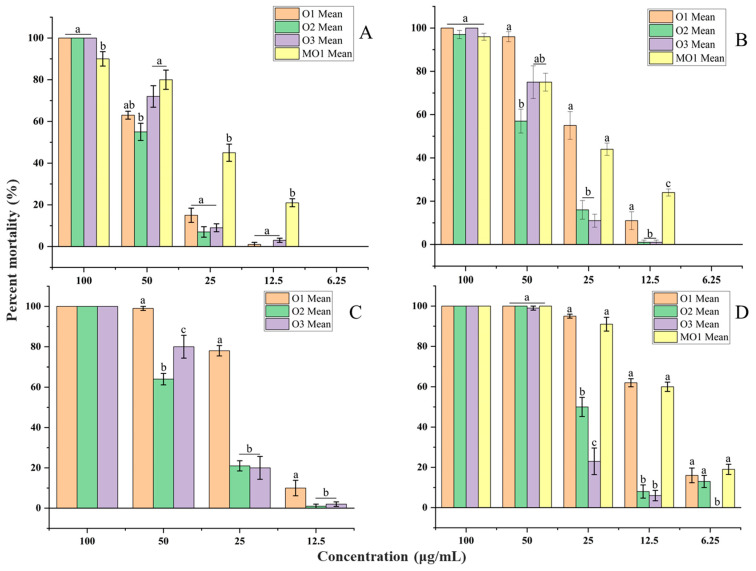
Mortality rate (%) of mosquito larvae at different concentrations of samples O1, O2, O3 and MO1 after 48 h of exposure, with mean ± SE. Values followed by the same letter (a–c) at each concentration are not statistically different at *p* < 0.05 as measured by Tukey’s test. (**A**) *Aedes aegypti*. (**B**) *Aedes albopictus*. (**C**) *Culex quinquefasciatus*. (**D**) *Culex fuscocephala*.

The essential oil samples exhibited larvicidal activity after 24 h of exposure, with 24 h LC_50_ values ranging from 43.53 to 53.46, 44.08 to 69.87, 27.25 to 44.34 and 11.73 to 31.97 µg/mL against *Ae. aegypti*, *Ae. albopictus*, *Cx. quinquefasciatus* and *Cx. fuscocephala*, respectively (Table 3). After 48 h of exposure, 48 h LC_50_ values ranged from 41.05 to 45.23, 23.04 to 38.89, 19.45 to 34.87, and 10.53 to 29.76 µg/mL against *Ae. aegypti*, *Ae. albopictus*, *Cx. quinquefasciatus* and *Cx. fuscocephala*, respectively (Table 3 and Table 4) (Figure 4 and Figure 5). MO1 microemulsion exhibited stronger toxicity than its essential oil, with 24 h LC_50_ values of 32.43, 29.81 and 11.16 µg/mL against *Ae. aegypti*, *Ae. albopictus*, and *Cx. fuscocephala*, respectively (Table 3 and Table 4). The larvicidal efficacy of sample O1 was significantly higher than that of O2 and O3 against *Ae. albopictus*, *Cx. quinquefasciatus*, and *Cx. fuscocephala* (*p* < 0.05). A similar pattern was noted for *Ae. aegypti*, though the difference did not reach statistical significance (*p* > 0.05) (Figure 4 and Figure 5). MO1 showed more stable activity at low concentrations than the essential oils (Figure 4 and Figure 5). Hung et al. (2022) described essential oils with 24 h LC_50_ values between 10 μg/mL and 50 μg/mL as very active and 24 h LC_50_ between 50 μg/mL and 100 μg/mL as moderately active [52]. Dias and Moraes (2014) and Pavela (2015) reviewed the larvicidal properties of essential oils and proposed that those exhibiting 24 h LC_50_ values below 100 µg/mL should be classified as active agents [53,54]. Therefore, the free essential oils and MO1 microemulsion formulation can be considered as potential mosquito larvicides.

Earlier investigations have demonstrated that (*E*)-β-caryophyllene possesses notable larvicidal activity against *Aedes aegypti* and *Aedes albopictus*, with 24 h LC_50_ values ranging from 56.87 to 70.80 µg/mL. In contrast, caryophyllene oxide showed comparatively weaker larvicidal effects against *Ae. aegypti*, with 24 h LC_50_ values reported between 127.9 and 136.6 µg/mL [55,56]. α-Humulene demonstrated larvicidal activity against *Ae. aegypti* and *Ae. albopictus*, with 24 h LC_50_ values ranging from 44.43 to 48.19 µg/mL and 31.49 to 43.86 µg/mL, respectively. In comparison, α-pinene exhibited stronger larvicidal effects against both species, with 24 h LC_50_ values between 12.94 and 23.05 µg/mL [56,57]. Germacrene D exhibited larvicidal activity against *Ae. aegypti*, *Ae. albopictus* and *Cx. quinquefasciatus* with 24 h LC_50_ values of 18.76–21.28 μg/mL, while β-pinene exhibited 24 h L_50_ values in the range of 27.69–32.23 g/mL [58]. Geijerene exhibited stronger larvicidal activity against *Ae. aegypti* and *Anopheles stephensi* than Germacrene D [59].

### 2.4. Repellent Activity

**Figure 6:**
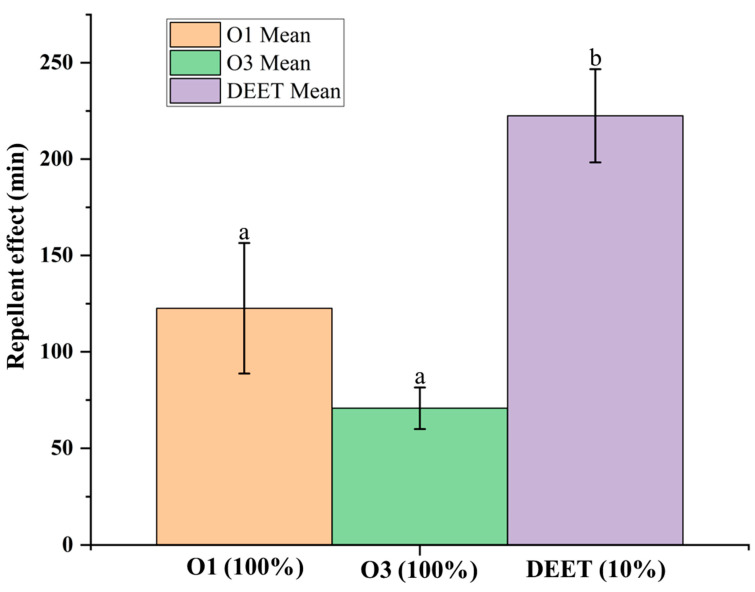
Repellent efficacy (mean ± SD) of *Chromolaena odorata* essential oils (O1 and O3) and DEET against *Aedes aegypti*. Bars with the same letters are not significantly different (*p* > 0.05, ANOVA followed by Tukey’s test).

The repellent activity of O1 against *Ae. aegypti* adults was higher than that of O3; however, the difference was not statistically significant (*p* > 0.05) (Figure 6). Essential oils are classified as highly effective repellents when their activity persists for more than 120 min, as reported in studies by Sritabutra et al. (2013) and Sutthanont et al. (2022) [60,61].

Some studies have reported that caryophyllene oxide and (*E*)-β-caryophyllene display repellent effects against *Ae. aegypti* and *An. quadrimaculatus* [62,63,64]. Germacrene D has been reported to exhibit repellent activity against *Myzus persicae* and *Acyrthosiphon pisum* [65]. Kiran and Pushpalatha (2013) reported that germacrene D and geijerene had strong repellent activity against adults of *Ae. aegypti* and *An. gambiae* [66]. Germacrene D and its mixture with α-pinene exhibited strong repellent activity against adults of *Ae. albopictus*, while the mixture of α-pinene and β-pinene exhibited weak repellent activity [67]. O1 contained a higher concentration of sesquiterpenoids compared to O3, whereas monoterpenoids were more abundant in O3. This compositional trend was also observed among the predominant sesquiterpenoid and monoterpenoid constituents. Such differences in chemical profiles may account for the variation in repellent efficacy observed between the O1 and O3 samples.

### 2.5. Fumigation Toxicity

O1 essential oil exhibited fumigant toxicity against *Ae. aegypti* at 0.5 and 24 h with LC_50_ values of 40.27% and 0.34%, respectively (Table 5). The essential oil of *Zingiber cassumunar*, which is composed predominantly of monoterpenoids (90.0%), has been shown to possess fumigant toxicity against *Ae. albopictus*, with LC_50_ values of 23.60% and 2.10% (*v*/*v*) following 0.5 and 24 h of exposure, respectively [68] (Li et al., 2021). Van et al. (2025) investigated the fumigant effect of *Zanthoxylum armatum* fruit essential oil, rich in monoterpenoids (95.11%), and found an LC_50_ of 0.52% after 1440 min of exposure [69]. In a similar context, Lucia et al. (2009) assessed the fumigant potential of essential oils extracted from 13 different *Eucalyptus* species against *Ae. aegypti*, applying an undiluted 10 µL dose and recording KT_50_ values between 4.19 and 12.03 min [70]. In this study, at 25% concentration, KT_50_ was 31.32 min (Table 6). Thus, the fumigant activity of *C. odorata* essential oil (O1) can be considered significant.

A study by Ma et al. (2020) reported that the compounds β-caryophyllene and α-pinene exhibited comparable fumigant activities, and both were more potent than caryophyllene oxide against *Megoura japonica*, *Plutella xylostella*, and *Sitophilus zeamais* [71]. β-Pinene was reported to have weaker fumigant activity against adults of *Lycoriella mali* than α-pinene [72]. Geijerene exhibited strong acute toxicity, while germacrene D exhibited moderate activity against *Spodoptera litura* [73].

### 2.6. Molluscicidal Activity

**Figure 7:**
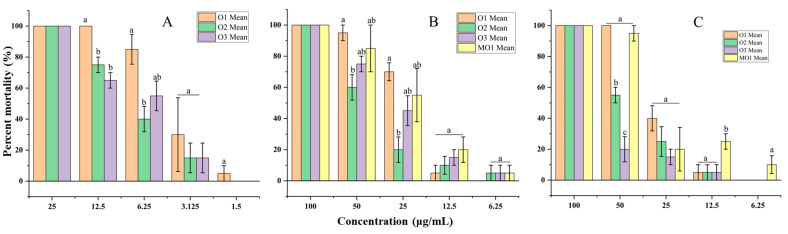
Mortality rate (%) of snails at different concentrations of samples O1, O2, O3, and MO1 with mean ± SE. Values followed by the same letter (a–c) at each concentration are not statistically different at *p* < 0.05 as measured by Tukey’s test. (**A**) *Physa acuta.* (**B**) *Indoplanorbis exustus.* (**C**) *Pomacea canaliculata*.

The essential oil samples O1, O2 and O3 were active against *P. acuta*, *I. exustus* and *P. canaliculata* with 48 h LC_50_ values in the ranges of 3.82–6.89, 21.88–26.97 and 25.80–54.38 µg/mL, respectively (Table 7). Essential oil O1 tended to exhibit stronger molluscicide activity than the two essential oil samples O2 and O3, as clearly shown by the mortality rate of snails at different concentrations (Figure 7). Microemulsion MO1 exhibited molluscicidal activity against *I. exustus* and *P. canaliculata* comparable to its free essential oil (O1), with 48 h LC_50_ values of 22.47 and 30.06 µg/mL, respectively (Figure 7).

Bacterial extracts derived from *Xenorhabdus* and *Photorhabdus* demonstrated molluscicidal activity against *I. exustus*, with 72 h LC_50_ values ranging from 89.81 to 100.32 μg/mL [74]. A cardiac glycoside-enriched fraction obtained from the leaves of *Nerium indicum* exhibited a 48 h LC_50_ of 20.12 μg/mL [74]. The methanolic extract of *Ambrosia artemisiifolia* produced a 24 h LC_50_ of 194 μg/mL, with sesquiterpene lactones identified as the active constituents, namely psilostachyin (LC_50_ of 15.9 μg/mL) and psilostachyin B (LC_50_ of 27.0 μg/mL) [75]. Additionally, three triterpenoids isolated from the methanol bark extract of *Eucalyptus exserta* displayed molluscicidal effects against *P. canaliculata*, with 72 h LC_50_ values of 27.3–42.6 μg/mL [76]. Thus, the essential oil samples O1, O2, and O3 and microemulsion MO1 can be compared with other biological molluscicides.

The sesquiterpenoids caryophyllene oxide, α-humulene, (*E*)-β-caryophyllene and α-pinene exhibited molluscicide activity with 48 h LC_50_ < 20 g/mL against *P. acuta*, *I. exustus* [57] and *P. canaliculata* [56].

### 2.7. AChE Inhibitory Activity

The O1 essential oil sample exhibited acetylcholinesterase inhibitory activity that was considered to be potent [77], with an IC_50_ value of 70.85 ± 5.47 µg/mL (Table 8). Thus, it is possible that the inhibition of acetylcholinesterase may be responsible for the insecticidal activities of this oil. In this study, α-pinene exhibited an IC_50_ value of 86.51 ± 6.24 µg/mL (0.64 ± 0.05 mM). Furthermore, this essential oil sample also shows potential for further research on aromatherapy to support the treatment of Alzheimer’s disease by inhibiting acetylcholinesterase [77]. The major constituents (*E*)-β-caryophyllene, β-pinene, and limonene were reported to have acetylcholinesterase inhibitory activity with IC_50_ values of 89.10, 71.45 and 53.16 µg/mL, respectively. The two compounds α-humulene and caryophyllene oxide exhibited IC_50_ values of 160.48 and 320.16 µg/mL, respectively [77]. (*E*)-β-Caryophyllene has been shown to inhibit acetylcholinesterase (AChE) from *Electrophorus electricus* (electric eel) by 32% at a concentration of 0.06 mM [78], while its inhibitory activity against AChE from human erythrocytes was reported with an IC_50_ value of 147 ± 15 µM [79]. Similarly, (*E*)-β-caryophyllene oxide demonstrated a 41.46 ± 2.66% inhibition of eel AChE at 200 µg/mL [80], and 35.0 ± 4.7% inhibition of bovine erythrocyte AChE at 250 µg/mL [81]. The essential oil of *Knema hookeriana* consisting of (*E*)-β-caryophyllene (26.2%), germacrene D (12.5%), δ-cadinene (9.2%), germacrene B (8.8%) and bicyclogermacrene (5.5%) exhibited an IC_50_ value of 70.5 µg/mL [82].

### 2.8. Antimicrobial Activity

The three essential oil samples exhibited significantly different antimicrobial activities. Among them, O2 demonstrated the highest potency, with IC_50_ values ranging from 0.53 to 3.17 µg/mL, whereas O1 showed the lowest activity, with IC_50_ values ranging from 9.34 to 42.56 µg/mL (Table 9). The essential oil of *Croton hirtus*, characterized by (*E*)-β-caryophyllene (32.8%), germacrene D (11.6%), β-elemene (9.1%), α-humulene (8.5%), and caryophyllene oxide (5.0%) as its major constituents, exhibited antimicrobial activity with IC_50_ values ranging from 3.12 to 5.98 μg/mL. Individually, (*E*)-β-caryophyllene, α-humulene, and caryophyllene oxide showed IC_50_ values in the ranges of 9.35–21.46 μg/mL, 3.24–10.45 μg/mL, and 2.67–12.56 μg/mL, respectively [83].

## 3. Materials and Methods

### 3.1. Plant Material

Fresh leaves of *C. odorata* were collected at the same time (June 2022) at three different locations, namely Da Nang (O1, GPS: 16°02′32″ N 108°08′09″ E), Quang Tri (O2, GPS: 16°46′28.4″ N 107°19′30.2″ E) and Quang Binh (O3, GPS: 17°18′08″ N 106°39′54″ E).

### 3.2. Hydrodistillation

Fresh leaves (O1: 5.0 kg; O2: 0.5 kg; O3: 1.0 kg) were chopped and subjected to hydrodistillation for 5 h using a Clevenger-type apparatus (Witeg Labortechnik, Wertheim, Germany). The essential oil yield was calculated as the mean of three replicates. The obtained oil was dried over anhydrous Na_2_SO_4_ and stored in sealed glass vials at 4 °C until further analysis and bioassays.

### 3.3. Gas Chromatographic Analysis

Gas chromatographic–mass spectral analysis and chiral gas chromatography–mass spectrometry were performed according to the protocol described by Pham et al. (2023) [84].

Gas chromatographic–mass spectral analysis used a Shimadzu GCMS-QP2010 Ultra system equipped with an electron impact (EI) ionization source (70 eV) (Shimadzu Scientific Instruments, Columbia, MD, USA). The mass spectrometer operated over a scan range of 40–400 *m*/*z* at a scan rate of 3.0 scans/s. Separation was achieved on a ZB-5ms capillary column (60 m × 0.25 mm, 0.25 μm film thickness) (Phenomenex, Torrance, CA, USA), with helium as the carrier gas at a constant flow rate of 2.0 mL/min and a column head pressure of 208 kPa. The injector, detector, and interface were maintained at 260 °C. The oven temperature was programmed from 50 °C to 260 °C at a rate of 2 °C/min. The essential oil was diluted in dichloromethane (1% *w*/*v*), and 1.0 μL was injected with a split ratio of 1:24.4. Percentages were calculated based on peak integration values. No corrections were carried out. Retention indices (RIs) were determined using a homologous series of *n*-alkanes, and compound identification was based on comparison of RI values and mass spectral fragmentation patterns with those in reference databases [85,86,87].

Chiral gas chromatography–mass spectrometry was carried out using a Shimadzu GCMS-QP2010S system operating in electron impact (EI) mode at 70 eV (Shimadzu Scientific Instruments, Columbia, MD, USA). Separation was achieved on a Restek B-Dex 325 chiral capillary column (Restek Corp., Bellefonte, PA, USA). The oven temperature was programmed from 50 °C to 120 °C at a rate of 1.5 °C/min, followed by an increase from 120 °C to 200 °C at 2.0 °C/min. A 0.1 μL aliquot of a 5% (*w*/*v*) essential oil solution in dichloromethane was injected with a split ratio of 1:45. Compound identification was based on comparison of retention times and mass spectral fragmentation patterns with those of authentic standards (Sigma-Aldrich, Milwaukee, WI, USA). Enantiomeric percentages were calculated directly from peak areas without correction or standardization.

### 3.4. Preparation of Microemulsion Formulas

The microemulsions (MEs) were formulated using the emulsion phase inversion (EPI) technique, as described by Giang et al. (2024) [88]. The essential oil (3%, *v*/*v*), 2-propanol (1%, *v*/*v*), and MCT oil (1%, *v*/*v*) were sequentially mixed and stirred for 15 min using a magnetic stirrer (H3770-HS, Benchmark, Sayreville, NJ, USA). Polysorbate 80 (10% *v*/*v*) was then added, followed by 30 min of stirring. Distilled water (85% *v*/*v*) was gradually introduced at 3 mL/min, and stirring continued until a transparent, homogeneous microemulsion (ME) formed. MEs were stored in transparent vials at 25 °C under a 12 h light/12 h dark cycle. Droplet size distribution was measured on Days 1 and 30 using dynamic light scattering (Zetasizer-Nano ZS, Malvern, UK).

### 3.5. Larvicidal Biassays

Larvicidal activities were performed as described by Van et al. (2025) [69]. *Aedes* spp. mosquitoes were continuously maintained at Duy Tan University, while egg rafts of *Cx. Quinquefasciatus* and *Cx. fuscocephala* were collected from domestic wastewater channels in Da Nang City. Eggs from all three mosquito species were hatched overnight in tap water; larvae were reared on cat food (Me O Tuna Adult Cat Dry Food), and the water was replaced daily. Adult mosquitoes were maintained on a 10% glucose solution and allowed to blood-feed on mice. All experiments were conducted under controlled conditions of 25 °C and 75 ± 5% relative humidity.

Essential oil (EO) solutions were prepared in ethanol (Merck) at concentrations of 100, 50, 25, 12.5, and 6.25 µg/mL, and 150 mL of each solution was transferred into 250 mL glass beakers. Subsequently, 25 third- and early fourth-instar larvae were transferred into beakers containing the test solution. Each concentration was tested in quadruplicate. Ethanol and permethrin served as negative and positive controls, respectively. Larval mortality was recorded after 24 and 48 h of exposure.

### 3.6. Repellency Bioassay

Female *Ae. aegypti* mosquitoes (4–5 days old), mated and starved for 24 h, were used in repellency assays under controlled laboratory conditions (25 ± 2 °C, 65–75% relative humidity). Mosquitoes were confined in 30 × 30 × 30 cm test cages.

Volunteers’ hands were washed with unscented soap, air-dried, and covered with plastic gloves, leaving a 30 cm^2^ window (5 × 6 cm) exposed on the dorsal side. Essential oil solutions were prepared in ethanol (Merck) at concentrations of 10%, 50%, and 100% (*v*/*v*). A 100 μL aliquot of each solution was applied to the exposed skin area and allowed to dry for 3 min. Ethanol served as the negative control, while 10% DEET in ethanol was used as the positive control.

For repellency testing, the treated hand was inserted into the mosquito cage for 5 min. Each concentration was tested in triplicate with a new cohort of mosquitoes per replicate. To assess protection duration, treated hands were exposed for 5 min at 15 min intervals until the first confirmed bite (defined as two bites during a single exposure or a second bite in the subsequent interval). Control hands were introduced before each trial to verify mosquito activity.

To ensure consistency, hands were held parallel to the vertical surface of the cage, as mosquitoes were observed to preferentially bite the underside when positioned perpendicularly. Complete protection time was recorded as the interval from application to the first confirmed biting event.

### 3.7. Fumigant Toxicity

Fumigant toxicity was evaluated following the protocol described by Van et al. (2025) [69]. Essential oil solutions were prepared in acetone at concentrations of 12.5%, 6.25%, 3.0%, 1.5%, 0.625%, and 0.15% (*v*/*v*). For each assay, a 1 × 3 cm strip of filter paper was impregnated with 10 µL of the test solution and suspended centrally in a 250 mL glass flask containing 15 non-blood-fed adult mosquitoes. Each concentration was tested in quadruplicate, with acetone alone used as the negative control. Mortality was recorded at 0.5, 1.0, 1.5, 2.0, 3.0, 4.0, 5.0, 6.0, and 24 h post exposure. Mosquitoes were considered dead if they failed to respond to a gentle agitation of the flask.

### 3.8. Molluscicidal Activity

Snail species collection and molluscicidal activity were performed as described by Luu et al. (2023) [57] and Pham et al. (2023) [84]. Snail individuals were collected from a natural environment, acclimatized to laboratory conditions (25 °C, 70 ± 5% RH) for 48 h and fed on leaves of *Lactuca sativa*. After the acclimatization period, healthy snails were used for the experiment. The essential oil was dissolved in ethanol to obtain a 1% (*w*/*v*) stock solution, and different volumes of the stock solution were dissolved in beakers containing 150 mL of distilled water to obtain a range of concentrations of 100, 50, 25, 12.5 and 6.25 µg/mL. Each concentration was repeated 4 times, 5 snails for each replication. After 24 h of exposure, the number of dead snails was recorded, and the test solution in the beakers was replaced with fresh distilled water, and the experiment was continued for another 24 h. At 48 h of exposure, the number of dead snails was recorded.

### 3.9. AChE Inhibitory Activity Assay

The protocol for assessing AChE inhibitory activity was performed as described by Ellman et al. (1961) [89]. Test samples, including essential oils and pure compounds, were initially dissolved in 100% dimethyl sulfoxide (DMSO) and subsequently diluted with deionized distilled water to obtain concentrations of 500, 100, 20, and 4 μg/mL. Each assay well contained 140 μL of phosphate buffer (pH 8.0), 20 μL of the test solution, and 20 μL of acetylcholinesterase (AChE) from electric eel (0.25 IU/mL), followed by incubation at 25 °C for 15 min. Subsequently, 10 μL of dithiobisnitrobenzoic acid (DTNB, 2.5 mM) and 10 μL of acetylthiocholine iodide (ACTI, 2.5 mM) were added, and the reaction was incubated for an additional 10 min at 25 °C. Absorbance was recorded at 405 nm. Galantamine served as the positive control, while the negative control lacked test samples. All assays were performed in triplicate.

### 3.10. Antimicrobial Activity

Antimicrobial activity testing protocols were performed as described by Luu-dam et al. (2023) [83]. The antimicrobial properties of the tested essential oils were evaluated using a serial dilution technique across multiple concentrations. Essential oils were initially dissolved in dimethyl sulfoxide (DMSO) and then serially diluted to yield final concentrations of 256, 128, 64, 32, 16, 4, and 2 µg/mL, with each concentration tested in triplicate. Microbial suspensions were adjusted to a final density of 2 × 10^5^ CFU/mL. The antimicrobial assays were conducted in sterile 96-well microtiter plates. A volume of 5.12 µL of each test solution (a stock concentration of 10 mg/mL) was transferred to the first well containing 100 µL of LB medium and then subjected to two-fold serial dilutions using 50 µL of medium per well, ultimately achieving a minimum test concentration of 2 µg/mL. Subsequently, 50 µL of microbial inoculum (2 × 10^5^ CFU/mL) was added to each well, and the plates were incubated at 37 °C for 24 h. Following incubation, absorbance at 650 nm was recorded using a microplate reader (Epoch, BioTek Instruments Inc., Winooski, VT, USA) to determine microbial growth. Streptomycin, kanamycin, tetracycline, nystatin, and cycloheximide (all procured from Sigma-Aldrich) served as positive control agents.

### 3.11. Statistical Analysis

For the agglomerative hierarchical cluster (AHC) analysis, the essential oil compositions of 37 samples of *C. odorata* reported in the literature along with the three samples in this study were used. The 40 essential oil compositions were used as operational taxonomic units and the percent compositions of the nine most abundant components (α-pinene, sabinene, β-pinene, 3-carene, cis-sabinene hydrate, linalool, 4,8,12-trimethyltrideca-1,3,7,11-tetraene (DMNT), cyclohexene-3,4-diethenyl-3-methyl, geijerene, geranial, pregeijerene, α-cubebene, β-cubebene, (*Z*)-β-farnesene, α-copaene, cyperene, (*E*)-β-caryophyllene, anisole, *trans*-verbenol, dauca-5,8-diene, γ-muurolene, germacrene D, *epi*-cubebol, bicyclogermacrene, cubebol, γ-cadinene, δ-cadinene, α-cadinene, β-copaen-4α-ol, α-elemol, naphthalene and caryophyllene oxide) were used to examine the chemical relationships between the *C. odorata* essential oils. Euclidean distance was used to determine dissimilarity and clustering was defined using Ward’s method. Principal component analysis (PCA) was carried out to provide a visual verification of the chemical relationships between the *C. odorata* essential oil samples using the major components (as above) as variables with a Pearson correlation matrix. The AHC and PCA were carried out using XLSTAT Premium v. 2018.1.1.62926 (Addinsoft, Paris, France).

## 4. Conclusions

The essential oils extracted from *C. odorata* collected in three central provinces of Vietnam exhibited highly similar chemical profiles, predominantly comprising α-pinene, geigerene, germacrene D, and (*E*)-β-caryophyllene. This particular chemotype demonstrated notable insecticidal, acetylcholinesterase inhibitory, and antimicrobial activities. These findings indicate that *C. odorata* holds considerable potential as a natural source for the development of essential oil-based biopesticides. Further investigations on additional chemotypes are warranted to identify those with the most potent pesticidal properties.

## Figures and Tables

**Table 1:** Chemical composition of *Chromolaena odorata* essential oil.

RI_calc_	RI_db_	Compound	%		
			O1	O2	O3
**927**	**927**	α-Thujene	tr	0.09	0.09
935	933	**α-Pinene**	**11.47**	**16.08**	**19.24**
950	950	Camphene	tr	tr	0.05
974	972	Sabinene	0.94	1.26	1.51
980	978	**β-Pinene**	**3.95**	**5.99**	**7.50**
991	991	Myrcene	0.67	0.65	0.89
1026	1025	*p*-Cymene	tr	0.08	0.15
1031	1030	Limonene	0.73	0.65	0.73
1037	1035	(*Z*)-β-Ocimene	0.13	0.13	0.13
1047	1046	(*E*)-β-Ocimene	1.00	0.94	1.08
1059	1058	γ-Terpinene	tr	tr	0.09
1135	-	*iso*-Geijerene	1.40	1.21	1.07
1145	1143	**Geijerene**	**10.55**	**9.71**	**8.96**
1286	1286	Cogeijerene	0.45	0.44	0.38
1295	1289	Pregeijerene	0.52	0.48	0.40
1333	1335	Bicycloelemene	0.13	0.12	tr
1337	1336	δ-Elemene	0.32	0.35	0.30
1348	1348	α-Cubebene	0.14	0.10	tr
1378	1377	**α-Copaene**	**5.26**	**4.45**	**4.02**
1385	1382	β-Bourbonene	0.17	0.30	0.19
1389	1387	β-Cubebene	0.16	0.10	0.06
1391	1390	*trans*-β-Elemene	1.74	1.39	1.30
1422	1418	**(*E*)-β-Caryophyllene**	**11.24**	**10.86**	**9.56**
1431	1433	β-Copaene	0.53	0.47	0.48
1435	1431	Dictamnol	1.56	1.94	2.15
1442	1438	*iso*-Dictamnol	0.35	0.19	0.20
1450	1453	*trans*-Muurola-3,5-diene	0.20	0.15	0.16
1457	1454	α-Humulene	3.22	2.97	2.97
1461	1458	*allo*-Aromadendrene	tr	0.10	0.08
1473	1473	*trans*-Cadina-1(6),4-diene	tr	0.25	0.26
1478	1478	γ-Muurolene	0.64	0.72	0.67
1484	1483	**Germacrene D**	**15.12**	**13.49**	**11.67**
1493	1492	*trans*-Muurola-4(14),5-diene	0.62	0.39	0.43
1497	1497	Bicyclogermacrene	2.42	2.10	1.72
1500	1500	α-Muurolene	0.96	0.78	0.75
1507	1504	(*E*,*E*)-α-Farnesene	tr	0.15	tr
1509	1508	β-Bisabolene	0.19	0.21	0.12
1514	1514	γ-Cadinene	0.54	0.32	0.36
1516	1515	Cubebol	0.41	tr	0.22
1521	1520	**δ-Cadinene**	**5.73**	**4.38**	**4.42**
1523	1527	*trans*-Calamenene	0.73	0.47	0.71
1525	1526	Zonarene	0.20	tr	tr
1533	1533	*trans*-Cadina-1,4-diene	0.13	tr	tr
1542	1541	α-Calacorene	0.62	0.56	0.60
1550	1549	α-Elemol	0.52	tr	tr
1552	1551	*iso*-Caryophyllene oxide	0.41	0.45	0.43
1559	1560	Germacrene B	0.41	0.46	0.48
1563	1562	(*E*)-Nerolidol	0.34	0.25	0.32
1579	1578	Spathulenol	0.76	0.73	0.74
1584	1587	**Caryophyllene oxide**	**5.02**	**4.40**	**5.33**
1590	1592	Globulol	tr	tr	0.25
1595	1594	Viridiflorol	0.35	0.39	tr
1610	1611	Humulene epoxide II	1.00	0.81	0.99
1617	1618	α-Corocalene	0.24	0.13	tr
1622	1625	Junenol	0.22	0.25	0.12
1628	1628	1-*epi*-Cubenol	0.10	0.48	0.35
1633	1629	*iso*-Spathulenol	0.26	tr	-
1638	1644	*allo*-Aromadendrene epoxide	-	0.35	0.38
1643	1643	τ-Cadinol	0.54	0.42	0.44
1645	1645	τ-Muurolol	0.44	0.43	0.43
1647	1651	α-Muurolol (=δ-Cadinol)	0.22	tr	tr
1650	1647	*cis*-Guaia-3,9-dien-11-ol	0.37	0.50	0.33
1656	1655	α-Cadinol	1.37	1.13	1.05
1674	1676	Mustakone	0.25	0.36	0.32
1716	1715	Pentadecanal	0.18	0.27	0.33
2108	2109	Phytol	1.09	1.39	1.08
		Monoterpene hydrocarbons	18.89	25.87	31.46
		Oxygenated monoterpenoids	0.00	0.00	0.00
		Sesquiterpene hydrocarbons	51.66	45.77	41.31
		Oxygenated sesquiterpenoids	12.58	10.95	11.70
		Diterpenoids	1.09	1.39	1.08
		Other	15.01	14.24	13.49
		Total identified	99.23	98.22	99.04

RI_calc_ = retention index determined with respect to a homologous series of *n*-alkanes on a ZB-5ms column. RI_db_ = reference retention index from the databases. tr = trace (<0.05%). Major components are highlighted in bold.

**Table 2:** Th enantiomeric distribution of monoterpenes in *Chromolaena odorata* essential oils and other members of the Asteraceae reported in the literature.

Compound	RT_db_	RT_exp_	O1	O2	O3	A.l.	A.a.	A.m.o.	E.n.
(−)-α-Pinene	15.92	15.47	54.5	58.3	54.8	50.6–88.0	99.3–99.4	72.8–87.3	59.5–90.6
(+)-α-Pinene	16.40	15.99	45.5	41.7	45.2	12.0–49.4	0.6–0.7	12.7–27.2	9.4–41.5
(+)-Sabinene	19.74	19.76	63.2	64.2	60.8	13.9–79.4	48.2–53.7	11.8–56.1	0.0–25.6
(−)-Sabinene	20.60	20.79	36.8	35.8	39.2	20.6–86.1	46.3–51.8	43.9–88.2	74.4–100.0
(+)-β-Pinene	20.27	20.10	95.8	96.9	96.7	2.5–25.8	12.1–13.5	1.1–18.8	0.4–10.4
(−)-β-Pinene	20.62	20.98	4.2	3.1	3.3	74.2–97.5	86.5–87.9	81.2–98.9	89.6–99.6
(−)-Limonene	25.06	25.46	30.9	38.7	39.6	38.0–100.0	50.2–60.4	31.8–83.1	40.2–95.8
(+)-Limonene	25.99	26.23	69.1	61.3	60.4	0.0–62.0	39.6–49.8	16.9–68.2	4.2–59.8

A.l.: *Artemisia ludoviciana* Nutt. A.a.: *Ambrosia acanthicarpa* Hook. [42]. A.m.o.: *Achillea millefolium* L. var. *occidentalis* DC. [43]. E.n.: *Ericameria nauseosa* (Pursh) G.L.Nesom & G.I.Baird [44].

**Table 3:** Larvicidal activity of *Chromolaena odorata* essential oil and its microemulsion at 24 h exposure.

Essential Oil	LC_50_ (95% Limits)	LC_90_ (95% Limits)	χ^2^	*p*
*Aedes aegypti*				
O3	43.53 (40.30–46.90)	68.96 (62.36–79.21)	4.46	0.216
O2	53.46 (50.31–57.58	71.26 (65.51–80.58)	2.92	0.404
O1	52.99 (49.58–57.25)	73.42 (67.43–82.42)	7.10	0.069
MO1	32.43 (28.68–36.79)	101.93 (83.00–133.89)	6.62	0.085
*Aedes albopictus*				
O3	53.42 (49.67–57.60)	83.71 (75.09–97.99)	2.07	0.557
O2	69.87 (63.94–76.81)	127.60 (110.95–155.54)	0.46	0.927
O1	44.08 (41.30–47.11)	61.89 (57.61–67.78)	1.59	0.663
MO1	29.81 (26.48–33.62)	87.62 (72.41–112.62)	8.11	0.044
*Culex quinquefasciatus*				
O3	44.34 (40.58–48.50)	83.06 (73.08–98.67)	14.94	0.002
O2	44.31 (41.08–47.85)	72.42 (65.20–83.30)	3.78	0.287
O1	27.25 (24.95–29.74)	52.65 (46.73–61.25)	7.35	0.061
MO1	Nt	Nt	Nt	Nt
*Culex fuscocephala*				
O3	31.97 (29.72–34.93)	41.69 (38.13–47.16)	3.56	0.313
O2	26.41 (24.31–28.89)	41.26 (37.54–46.57)	4.95	0.176
O1	11.73 (10.60–12.92)	24.55 (21.40–29.53)	0.91	0.823
MO1	11.16 (10.12–12.30)	23.95 (20.87–28.61)	2.14	0.710

MCO1: Microemulsion of CO1 essential oil sample. Nt: not tested.

**Table 4:** Larvicidal activity of *Chromolaena odorata* essential oil and its microemulsion at 48 h exposure.

Essential Oil	LC_50_ (95% Limits)	LC_90_ (95% Limits)	χ^2^	*p*
*Aedes aegypti*				
O3	45.23 (42.37–48.42)	63.82 (59.27–70.19)	1.68	0.642
O2	46.27 (42.96–49.75)	72.12 (65.23–83.16)	4.61	0.203
O1	41.05 (37.74–44.62)	71.37 (63.63–83.23)	5.17	0.160
MO1	28.54 (25.31–32.22)	85.72 (70.68–110.50)	7.55	0.056
*Aedes albopictus*				
O3	38.89 (35.94–42.01)	61.78 (55.88–70.63)	1.97	0.578
O2	43.43 (39.73–47.52)	81.75 (71.90–97.09)	3.17	0.366
O1	23.04 (21.18–25.03)	40.49 (36.08–47.23)	2.23	0.527
MO1	27.08 (24.13–30.40)	75.52 (63.11–95.53)	10.59	0.014
*Culex quinquefasciatus*				
O3	34.87 (32.12–37.87)	58.93 (52.72–68.31)	1.85	0.603
O2	39.07 (35.80–42.65)	71.37 (63.17–83.91)	7.55	0.056
O1	19.45 (18.06–20.93)	30.66 (27.84–34.82)	1.27	0.736
MO1	Nt	Nt	Nt	Nt
*Culex fuscocephala*				
O3	29.76 (27.70–32.38)	40.52 (37.09–45.63)	5.31	0.257
O2	24.32 (22.34–26.63)	38.83 (35.26–43.96)	13.90	0.003
O1	10.53 (9.57–11.54)	20.21 (17.79–24.02)	0.90	0.825
MO1	9.84 (8.81–10.97)	25.09 (21.45–30.68)	13.11	0.011

MO1: Microemulsion of O1 essential oil sample. Nt: not tested.

**Table 5:** Knockdown time of *Aedes aegypti* mosquito exposed to *Chromolaena odorata* essential oil (O1).

Concentration (%)	KT_50_ (m)	KT_90_ (m)	χ^2^	*p*
25	31.32 (27.60–35.16)	63.42 (54.97–76.54)	14.08	0.080
12.5	40.76 (35.51–46.10)	98.86 (85.27–119.11)	19.71	0.011
6.25	54.87 (48.10–61.73)	139.45 (120.57–167.47)	22.99	0.003
3.0	212.04 (186.54–243.40)	824.78 (639.34–1170.59)	8.79	0.360
1.5	550.24 (456.51–697.94)	2392.87 (1644.53–4098.97)	4.02	0.855
1.0	641.95 (534.11–811.29)	2306.43 (1634.53–3770.21)	13.06	0.110
0.5	1081.33 (873.80–1422.51)	3607.33 (2500.65–6138.99)	7.87	0.446

**Table 6:** Adulticidal activity of *Chromolaena odorata* essential oil (O1) on *Aedes aegypti*.

Time (min)	LC_50_ (%)	LC_90_ (%)	χ^2^	*p*
15	106.03 (47.59–641.77)	1469.91 (320.34–54115.90)	3.72	0.590
30	40.27 (26.48–81.72)	314.51 (134.50–1512.64)	3.73	0.590
60	8.64 (7.35–10.28)	28.87 (22.17–41.64)	5.94	0.312
90	4.68 (4.05–5.43)	12.95 (10.49–17.16)	9.91	0.078
120	3.59 (3.15–4.09)	8.08 (6.75–10.31)	18.23	0.003
180	2.69 (2.39–3.05)	5.72 (4.82–7.25)	17.77	0.003
240	2.36 (2.07–2.69)	5.57 (4.62–7.19)	11.80	0.038
300	1.95 (1.71–2.23)	4.88 (4.02–6.38)	10.50	0.062
360	1.85 (1.63–2.12)	4.63 (3.81–6.06)	8.71	0.121
1440	0.34 (0.19–0.47)	1.44 (1.13–2.08)	3.42	0.636

**Table 7:** Molluscicidal activity of *Chromolaena odorata* essential oils (µg/mL).

Essential Oil	LC_50_ (95% Limits)	LC_90_ (95% Limits)	χ^2^	*p*
*Physa acuta*				
O3	6.89 (5.33–8.84)	17.30 (12.72–29.43)	5.75	0.331
O2	7.16 (5.62–9.07)	16.26 (12.21–26.90)	2.34	0.800
O1	3.82 (3.09–4.71)	6.95 (5.47–11.13)	0.57	0.989
CuSO_4_ (positive control)	0.66 (0.55–0.80)	0.85 (0.72–1.17)	0.00	0.998
*Indoplanorbis exustus*				
O3	26.97 (20.97–35.00)	68.70 (49.60–123.03)	1.77	0.779
O2	38.57 (29.97–50.74)	98.63 (69.98–188.00)	5.73	0.220
O1	21.88 (18.01–26.24)	35.22 (28.81–53.59)	1.12	0.878
MO1	22.47 (17.41–28.90)	55.14 (40.29–98.39)	0.70	0.872
CuSO_4_ (positive control)	0.28 (0.23–0.33)	0.43 (0.35–0.64)	0.3618	0.948
*Pomacea canaliculata*				
O3	54.38 (43.49–69.87)	112.15 (83.46–207.92)	11.40	0.022
O2	39.50 (31.47–50.12)	83.49 (62.79–143.75)	3.03	0.552
O1	25.80 (21.75–30.90)	38.82 (32.04–64.33)	1.79	0.774
MO1	30.06 (24.64–49.72)	49.72 (41.08–66.80)	7.15	0.128
Positive control (tea saponin)	24.78 (23.26–26.72)	32.62 (29.98–37.10)	0.1301	0.988

**Table 8:** Acetylcholinesterase inhibitory activity of *Chromolaena odorata* essential oil (O1).

Concentration (µg/mL)	O1		Concentration (µg/mL)	Galanthamine	
	Inhibition (%)	SD		Inhibition (%)	SD
500	91.72	2.77	10	91.07	1.31
100	59.48	1.54	2	56.56	1.69
20	25.38	1.15	0.4	21.83	0.93
4	8.93	0.62	0.08	9.07	0.42
IC_50_	70.85 ± 5.47		IC_50_	1.70 ± 0.12	

**Table 9:** Antimicrobial activity of *Chromolaena odorata* essential oil.

Microorganism	Essential Oil (µg/mL)						Positive Control (µg/mL)			
	O1		O2		O3		Streptomycin		Cyclohexamide	
	MIC	IC_50_	MIC	IC_50_	MIC	IC_50_	MIC	IC_50_	MIC	IC_50_
*Enterococcus faecalis* ATCC299212	32	9.34 ± 1.46	2	0.67 ± 0.01	16	5.34 ± 1.32	256	50.34 ± 2.32	Nt	Nt
*Staphylococcus aureus* ATCC25923	64	19.45 ± 2.13	2	0.54 ± 0.02	32	12.45 ± 0.05	256	45.24 ± 1.36	Nt	Nt
*Bacillus cereus* ATCC14579	64	21.25 ± 0.23	8	3.17 ± 0.78	32	9.45 ± 0.17	128	20.45 ± 0.39	Nt	Nt
*Escherichia coli* ATCC25922	128	42.56 ± 2.56	2	0.53 ± 0.45	32	9.76 ± 1.32	32	9.45 ± 0.35	Nt	Nt
*Pseudomonas aeruginosa* ATCC27853	Na	Na	Na	Na	Na	Na	256	64.67 ± 1.89	Nt	Nt
*Salmonella enterica* ATCC13076	64	40.34 ± 3.21	8	3.23 ± 0.06	32	9.24 ± 0.74	128	45.67 ± 2.30	Nt	Nt
*Candida albicans* ATCC10231	32	20.45 ± 1.04	2	0.67 ± 0.021	32	9.27 ± 0.96	Nt	Nt	32	10.46 ± 0.32

Na: not active; Nt: not tested.

## Data Availability

Data will be made available on request.

## Supplementary Materials

The following supporting information can be downloaded at https://www.mdpi.com/article/10.3390/molecules30173602/s1, Table S1: A review of the literature on the chemical composition of *Chromolaena odorata* essential oil.
